# Suprarenal Extract as a Hæmostatic

**Published:** 1902-02-08

**Authors:** 


### SUPRARENAL EXTRACT AS A HÆMOSTATIC.

THE utility of suprarenal extract as a hæmostatic is now well established. It has, however, been doubted by some whether it is effective in cases of hæmophilia, seeing that this condition is regarded as due to congenital hypoplasia of the muscular coats of the arteries. Dr. William Milligan,[1] however, relates a case in which hæmorrhage came on after removal of naso-pharyngeal adenoids in a boy aged 10, who happened to be a hæmophilic, this fact not being known at the time of operation. The amount of bleeding at the operation had been comparatively slight, but about 20 hours afterwards the boy vomited several clots of blood of a peculiar pale colour. Examination revealed a constant though slow oozing of a pale pinkish fluid from the naso-pharynx along the posterior pharyngeal wall. The nose was syringed with iced water and a hypodermic injection of ergotine was given. As the oozing continued on the following day packing with iodoform gauze was tried. This failing the plug was removed and another soaked in turpentine was introduced, but with no better result. Finally a plug of gauze soaked in a solution of suprarenal extract was tightly packed into the naso pharynx. No further hæmorrhage occurred and the patient made an excellent though slow recovery.

[1] Brit. Med. Jour., Feb. 1.

